# What Is Wrong with St. George's Hospital?

**Published:** 1913-12-06

**Authors:** 


### What is Wrong with St. George's Hospital?

A CORRESPONDENT'S POINT OF VIEW.

A successful hospital administrator with some experience writes:

It is difficult to understand the aims and objects of those who are acting for the Governors of St. George's Hospital. That they have had a rough time of it since the retirement of Mr. Todd does not necessarily show a diminution in interest in the welfare of the hospital, but probably a lack of confidence in the way the affairs of the hospital are carried on. No sooner had the old hand retired than the Governors began to make experiments. They started off by appointing a soldier to do the secretary's work. It is reported that as a military man he was exceptionally able, but as a secretary the same could not be said, and two years was enough. Then they appointed a medical superintendent with administrative duties. Mr. Friend was too good a doctor to make a good secretary. As a medical man he was good; as a secretary he was an amateur.

Now it is rumoured that it is the intention of the Governors to appoint a third amateur—this time it is to be an architect. Let us hope for the sake of St. George's that he is not too good an architect, then perhaps he may spare time from his profession to excel as an administrator. We cannot serve God and mammon. But surely St. George's Hospital is important enough to have at the head of its affairs a business man—a trained hospital secretary. The treasurer up to the present has not had a chance. He has had to face year by year a diminishing income and a recurring deficit in the one hospital in London that it is easiest to finance. He has had no expert at hand to guide him in getting in funds, and no one who knows how to effect economies without its being known that economies are being effected.

Take this glance at the financial position since the office of secretary, in the ordinary sense of the word, has been abolished. Annual subscriptions have fallen from £6,500 to £5,000. In some years donations have decreased to two-thirds of their normal amount, and other sources of income show a lack of attention. In the last decade the expenditure has exceeded the income by over £30,000, although in 1907 more than £63,000 was received in legacies. At the present rate of expenditure St. George's wants about £16,000 a year in legacies to keep it going. It wants a man who knows something of the work to make up this leeway.

But that is not all, the impasse between the King's Fund and the hospital ought never to have occurred, most probably it never would have occurred had a competent and tactful secretary conducted the business under the direction of a committee. Still the present is a critical time. The amalgamation with Westminster has to be carried through; much laborious and careful work has to be done, and who is to do it? Mr. Quennell is not likely to desire to go through the stress of increasing work, to attend the constant committees that will be necessary before the amalgamation is complete. Perhaps, after all, the Governors will compromise and set up a triple control, consisting of a soldier, a doctor, and an architect to carry on the secretarial duties. We shall see. There is great work to be done and great work sometimes brings forth strong men. St. George's will worry through somehow, but let us hope that a great opportunity will not be lost for the want of a little common sense.

